# Construction and In Vitro Evaluation of Brain-Targeted Lutein Liposomes

**DOI:** 10.3390/foods14213611

**Published:** 2025-10-23

**Authors:** Tingting You, Zhiguo Na, Ruobing Zhao, Yongqiang Ma

**Affiliations:** School of Food Engineering, Harbin University of Commerce, Harbin 150028, China; ytt_happiness@163.com (T.Y.); nazhiguo9933@163.com (Z.N.); zhaoruobing1996@163.com (R.Z.)

**Keywords:** lutein, liposome, brain-targeting, release in vitro, blood–brain barrier

## Abstract

Lutein is one of carotenoids in the human brain that is consistently associated with all cognitive performance indicators, and its levels are closely linked to age-related cognitive decline. However, lutein application is limited by its poor stability and low bioaccessibility. In this study, a lutein-loaded delivery system was developed to enhance stability and achieve brain-targeting effects. Using high-speed shear and ethanol hydration methods, PEGylated lutein liposomes with lactoferrin (Lf-LLips) were constructed and characterized. The morphology was observed using TEM and AFM. Particle sizes and lutein retention rates were evaluated under different temperatures (4 °C, 25 ± 2 °C, 50 °C), light (diffusion light, DL; light shielding, LS), and storage durations at 28 d. Compared with free lutein, the in vitro release behavior and permeability across the blood–brain barrier of the systems were investigated. Lf-LLips exhibited a particle size of 186.63 ± 2.04 nm and a potential of −30.53 ± 1.65 mV, and the lutein encapsulation efficiency was 83.11 ± 1.67%. When stored under LS, the particle size of Lf-LLips remained under 190 nm at 4 °C for 28 days, and the retention rate of lutein exceeded 80%. The release curve of Lf-LLips in vitro over 72 h followed the Weibull model. Furthermore, the permeability across the blood–brain barrier model within 12 h was 22.73 ± 1.42%. These results demonstrate that Lf-LLips significantly improve the stability of lutein and exhibit sustained-release properties along with brain-targeting efficiency. The findings demonstrate the promising future of lutein for applications in brain health enhancement.

## 1. Introduction

With the accelerating aging of the population, the incidence of neurodegenerative diseases—primarily triggered by brain aging—has risen sharply [1]. These diseases are irreversible and incurable [2]. After their onset, patients can only rely on medication for their whole life, imposing a heavy burden on families, society, and the patients themselves. Recent studies have shown that nutritional interventions can delay or ameliorate symptoms of brain aging [3,4,5,6,7,8], such as supplementation with omega-3 fatty acids, vitamin E, and carotenoids. However, the brain is an organ that is rich in lipids and metabolically active, and its unique blood–brain barrier (BBB) maintains neural homeostasis [9]. Simultaneously, more than 98% of small molecules and virtually all biologics are blocked from entering the brain. Hence, the delivery of neuroprotective drugs and nutrients faces three major hurdles: (i) inability to cross the intact BBB, (ii) inability to retain drugs and nutrients under conditions of BBB disruption [10], and (iii) high toxicity of synthetic carriers [11]. In summary, developing novel delivery systems that can traverse the BBB, accumulate effectively within the brain, and exert therapeutic effects has become one of the most formidable challenges in modern brain-health research.

Lutein, an oxygen-containing carotenoid characterized by its conjugated polyene structure, is one of the carotenoids persistently associated with cognitive performance indicators in the human brain [12,13]. It exerts neuroprotective effects through multiple mechanisms, including antioxidant [14] and anti-inflammatory activities [15], and it inhibits Aβ aggregation [16,17,18]. Epidemiological studies have shown a positive correlation between dietary lutein intake and cognitive function, with its concentrations in the cerebral cortex and hippocampus being negatively associated with age-related cognitive decline [19,20]. However, due to its lipophilic nature [21] and poor chemical stability, the bioavailability of lutein following oral or intravenous administration is low [22], severely limiting its application in the treatment of brain disorders. Liposomes offer advantages that include high drug-loading capacity [23,24], good biocompatibility [25,26,27,28], and potential for surface functionalization [29,30,31]. Nevertheless, conventional liposomes are prone to rapid clearance by the reticuloendothelial system (RES) [32], resulting in a short half-life in vivo that hinders their applicability for brain delivery. Since the 1990s, surface modification with hydrophilic polymers such as polyethylene glycol (PEG) has been employed to significantly prolong systemic circulation time [33,34] and leverage the enhanced permeability and retention effect at pathological sites (e.g., tumors or inflamed tissues) for passive targeting [35,36,37]. Currently, PEGylated doxorubicin liposomes (Doxil) have been approved for clinical cancer therapy, demonstrating a clear advantage in reducing cardiotoxicity and improving therapeutic efficacy [38]. Building on this, covalent conjugation of ligands such as lactoferrin [39], transferrin [40], or Angiopep-2 [41] can markedly enhance brain delivery via receptor-mediated transcytosis. Currently, several targeted delivery systems for carotenoids [42,43], such as β-carotene, astaxanthin, and lycopene, have been developed using the polymer nanoparticles [44], solid lipids [45], and nanoemulsions [46]. However, studies have not yet reported the application of PEGylation to lutein-based delivery systems. In this study, lactoferrin-conjugated PEGylated lutein liposomes (Lf-LLips) were prepared using high-speed shear and ethanol hydration methods and systematically characterized. The stability of Lf-LLips was evaluated under various temperatures, light conditions, and storage durations. In addition, their release behavior in vitro and brain-targeting efficacy were investigated. This research aims to provide a viable delivery system in the field of improving brain health, with considerable potential for broader impact.

## 2. Materials and Methods

### 2.1. Main Materials

Lutein (≥90%) was purchased from Yuan Sheng Tai Bio-tech Co., Ltd. (Tianjin, China). Egg yolk lecithin (≥85%), cholesterol (≥98%), 1-Ethyl-3-(3-dimethylaminopropyl) carbodiimide (analytical grade), methanol and methyl tert-butyl ether (HPLC grade), and BHT (analytical grade) were obtained from Shanghai Macklin Biochemical Technology Co., Ltd. (Shanghai, China). N-Hydroxysuccinimide (analytical grade) was sourced from Aladdin Scientific. Distearoylphosphatidylethanolamine-PEG (2 kDa, analytical grade) and DSPE-PEG-COOH (analytical grade) were supplied by YuSi Medical Technology (Chongqing, China).

### 2.2. Preparation of Lactoferrin-Conjugated PEGylated Liposomes for Lutein Loading (Lf-LLips)

A lutein-loaded delivery system was prepared using high-speed shear combined with the ethanol hydration method. An appropriate amount of egg yolk lecithin and cholesterol was weighed, all dissolved in the anhydrous ethanol. Then, the mixture was kept in light-protected conditions for 2 min before being homogenized via high-speed shear. Meanwhile, an appropriate amount of ultrapure water was preheated in a 40 °C water bath. The mixed lutein-ethanol solution was slowly dripped into the ultrapure water under constant-temperature magnetic stirring to facilitate hydration. The ethanol was removed via rotary evaporation for 5 min to obtain liposomes (BLips). Subsequently, PEGylation was accomplished via the post-insertion technique. Specifically, the pre-formed plain liposomes were incubated with DSPE-PEG_2000_ of 5% in a 50 °C water bath for 1 h to facilitate the efficient insertion of DSPE-PEG_2000_ molecules into the lipid bilayer. EDC, NHS, and DSPE-PEG_2000_-COOH were then added at a molar ratio of 10:10:1 and stirred at room temperature for 15 min. An excess amount of lactoferrin was introduced, followed by incubation in a 37 °C water bath for 3 h. The mixture was dialyzed overnight to obtain lactoferrin-conjugated PEGylated liposomes for lutein loading (Lf-BLips). To investigate the effects of the egg yolk lecithin-to-cholesterol ratio (2:1, 3:1, 4:1, 5:1, 6:1, 7:1, *m*/*m*), ethanol-to-water ratio (1:5, 2:5, 3:5, 4:5, 5:5, 6:5, *v*/*v*), shear speed (6000, 8000, 10,000, 12,000, 14,000, 16,000, r/min), and shear time (1, 3, 5, 7, 9, 11, min) on the particle size of the Lf-BLips. After optimization using the Central Composite Design–Response Surface Methodology (CCD—RSM) as Table 1, the system was applied to lutein loading. And the influence of lutein addition on the particle size and encapsulation efficiency was studied to determine the optimal preparation process for Lf-LLips. The prepared Lf-LLips were stored at 4 °C protected from light.

### 2.3. Determination of Lutein Content

First, 100 μL of the prepared liposome was accurately pipetted, methanol was added, vortexing was conducted for 30 s to disrupt the vesicles, and then centrifugation was conducted at 10,000 r/min for 3 min; this cycle was repeated three times. The supernatant was collected and passed through a membrane filter of 0.22 μm, and the filtrate was stored for analysis. The lutein content in the liposomes was determined with HPLC using a C30 column (5 µm) maintained at 30 °C. A gradient elution with methanol/water (88:12, *v*/*v*, containing 0.1% BHT) and methyl tert-butyl ether (containing 0.1% BHT) was applied at a flow rate of 1.0 mL/min. The wavelength was set to 445 nm, and there was an injection of 50 µL per sample.

### 2.4. Determination of Lactoferrin Content

First, 100 μL of the liposome was pipetted into a micro-tube, and then 200 µL each of methanol, chloroform, and distilled water were sequentially added. This was followed by vortexing for 30 s and then centrifugation at 10,000 r/min for 3 min. The upper phase was carefully removed, and the protein remained. The pellet was dissolved in 50 µL of ultrapure water and held for analysis. The BCA working reagent was prepared according to the kit’s instructions; it was stable for 24 h at room temperature. The following day, the protein standard was diluted to generate a calibration curve. Then, 20 µL of each standard or the solubilized sample was dispensed into a 96-well plate, 200 µL of BCA working reagent was added per well, and incubated at 60 °C for 1 h in the dark. The absorbance at 562 nm shows on a microplate reader. The concentration of lactoferrin covalently linked to liposomes was calculated from the standard curve, and the junctional efficiency (JE) was determined using Equation (1).

JE(%) = C*_j_*/C*_T_* × 100 (1)

where C*_j_* represents the amount of junctional lactoferrin in Lf-LLips, while C*_T_* represents the total amount of lactoferrin added.

### 2.5. Determination of Particle Size, Zeta Potential, and the Polydispersity Index

The sample was diluted tenfold with distilled water and filtered using 0.45 μm for subsequent analysis. The particle size (*d*), potential (ζ), and polydispersity index (PDI) were measured using a Malvern Zetasizer (Malvern, UK).

### 2.6. Determination of Lutein Encapsulation Efficiency

Firstly, free lutein of Lf-LLips was removed at 3000 rpm for 10 min. To collect a certain amount of the supernatant, 2.0 mL of methanol was added, and this was sonicated for 10 min to disrupt the liposomes. The lutein-loaded content in the Lf-LLips was then determined, and the encapsulation efficiency (EE) of lutein was calculated according to Equation (2).

EE(%) = C*_i_*/C*_T_* × 100 (2)

where C*_i_* represents the lutein-loaded content in Lf-LLips, while C*_T_* represents the total lutein content in Lf-LLips.

### 2.7. Transmission Electron Microscopy (TEM) Analysis

A 5 μL aliquot of the sample was placed onto a carbon-coated copper grid for 3–5 min. To wick away excess liquid with filter paper, and then 2% phosphotungstic acid (5 μL) was added; this was allowed to stand for 2–3 min. The remaining stain was removed, and the grid was allowed to air-dry at room temperature before imaging the morphology via the transmission electron microscopy (TEM).

### 2.8. Atomic Force Microscopy (AFM) Analysis

The liposomes were scanned in both 2D and 3D modes to obtain nanometer-scale topographic images through AFM. To fix the specimen on the sample stage, a cantilever was installed. The laser was adjusted to the optimal position by monitoring the sum and deflection signals.

### 2.9. Stability

The particle size (*d*) of Lf-LLips and the retention rate (RR) of lutein were evaluated at different temperatures (4 °C, 50 °C and room temperature) and light (diffusion light, light shielding) over 7 days, as well as for a storage duration of 28 days. The RRs of lutein were calculated according to Equation (3).

RR(%) = C*_t_*/C_0_ × 100 (3)

where C*_t_* represents the lutein content in the Lf-LLip delivery system every two days or every week, while C_0_ represents the initial lutein content in the Lf-LLip delivery system.

### 2.10. Release Characteristics In Vitro

The release characteristics of Lf-LLip in vitro were investigated using a dialysis method. A 1.0 mL aliquot of Lf-LLips was placed into a pre-activated dialysis bag of MWCO with 8.0–14.0 kDa, which was then immersed in a beaker containing 100 mL of PBS (pH 7.4) with 0.5% Tween-80. The beaker was placed in a constant-temperature shaker that was maintained at 37 °C and stirred at 100 rpm. At predetermined time points (1, 2, 4, 8, 12, 24, and 48 h), 1.0 mL of the release medium was collected for the quantification of lutein concentration, and an equal volume of fresh release medium was replenished to continue the incubation. The cumulative release rate (CRP) was calculated according to Equation (4).

(4) $$\mathrm{CRP}\left(\%\right)=(C_{i}V_{t}+V_{i}\sum_{n=1}^{i-1}C_{i})/m_{t}\times 100$$

where m*_t_* is the total amount of lutein added to the sample; V*_t_* is the total volume of the PBS solution, which is 100 mL; C*_i_* is the concentration of lutein in the release medium withdrawn at each time; and V*_i_* is 1.0 mL.

### 2.11. Brain Targeting In Vitro

The in vitro blood–brain barrier (BBB) model was established using brain microvascular endothelial cells (bEnd.3) and cerebellar astrocytes (C8-D1A) from mice to evaluate brain-targeting activity. Both cell lines were maintained in DMEM supplemented with 10% fetal bovine serum (FBS) and cultured at 37 °C, 5% CO_2_, and a relative humidity of 90%. When cultures reached 90% confluence in the logarithmic growth phase, the cells were passaged. The following procedures were performed after 30 min of UV sterilization of the biosafety cabinet. The spent culture medium was removed and gently rinsed with PBS. After removing the PBS, 0.25% trypsin-EDTA was added. The cell culture was swirled in a “figure-eight” motion to ensure complete coverage. Upon detachment, cells were resuspended in complete medium, triturated to a single-cell suspension, and enumerated under a microscope using a hemocytometer.

#### 2.11.1. In Vitro Cytotoxicity

The bEnd.3 and C8-D1A cells were harvested and adjusted to a density of 5 × 10^4^ cells/mL in the logarithmic growth phase. A 100 μL aliquot of the cell suspension was added to each well of a 96-well plate and incubated for 18 h to allow cell attachment. After removing the culture medium, the cells were treated with complete medium containing either LLips or Lf-LLips at various concentrations. The groups without LLip or Lf-LLip treatment were used as the control. Then, the plates were incubated for 24, 48, and 72 h. Following the CCK-8 kit’s instructions, CCK-8 (10 μL) reagent was added, and the plates were further incubated for 2 h under the same conditions. The values of optical density (OD) were measured at a wavelength of 450 nm. The relative cell viability (RCV) was calculated according to Equation (5)

RCV(%) = (OD*_i_* − OD*_c_*)/(OD_0_ − OD*_c_*) × 100 (5)

where OD*_c_* is the OD value of the control; OD*_i_* is the OD value at 24, 48, and 72 h; and OD_0_ is the initial OD value.

#### 2.11.2. Establishment of the BBB Co-Culture Model

Log-phase C8-D1A astrocytes were detached with 0.25% trypsin-EDTA, resuspended in complete medium, and counted. The suspension was adjusted to 5 × 10^5^ cells mL^−1^. Then, 100 μL of the cell suspension was added to the bottom side of a 12-well Transwell plate. The inserts were inverted and incubated for 8 h (37 °C, 5% CO_2_, 90% RH) to allow firm adhesion. After 8 h, bEnd.3 microvascular endothelial cells were trypsinized in the logarithmic growth phase, counted and diluted to 5 × 10^4^ cells mL^−1^. Eight hundred microliters of bEnd.3 were added to the 12-well Transwell inner chamber. Subsequently, 1.5 mL complete medium was added to an outer 12-well Transwell. The co-culture was maintained in an incubator. The values of the transepithelial electrical resistance (TEER) were monitored at 12 h intervals. Once the TEER value stabilized and reached a plateau, it indicated the successful establishment of the BBB model.

#### 2.11.3. Permeability

After the model was established, the original culture medium was removed and replaced with complete medium containing either Lf-LLips or LLips at a concentration of 2.5 mM. The cells were further incubated for 12 h, during which the TEER value was continuously monitored. The solution at the bottom of the Transwell was collected at 1, 2, 4, 8, and 12 h. Methanol was added to demulsify the samples and to measure the lutein content. The permeability (PA) of lutein was calculated according to Equation (6).

PA(%) = C*_t_*/C*_i_* × 100 (6)

where C*_t_* is the lutein content at 1, 2, 4, 8, and 12 h, while C*_i_* is the initial lutein content.

### 2.12. Statistical Analysis

The results of all experiments were expressed as the mean ± standard deviation (*n* = 3) and performed in triplicate. Data were processed using the GraphPad Prism software (version 10.0). Statistical analysis was carried out using the analysis of variance, and a *p*-value was less than 0.05 that was considered to indicate statistical significance.

## 3. Results and Discussion

### 3.1. Optimization of Lactoferrin-Conjugated PEGylated Liposomes for Lutein Loading (Lf-LLips) Preparation Process and Characterization

#### 3.1.1. Optimization of Lf-BLips Preparation Conditions

**Figure 1:**
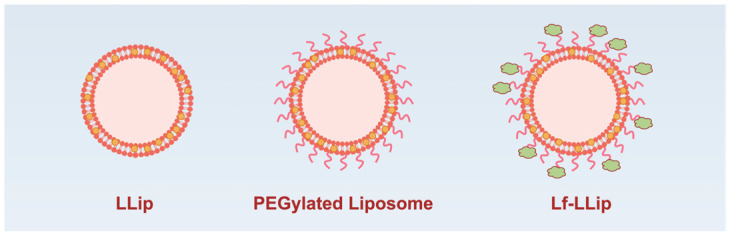
A schematic illustration of the lutein delivery system Lf-LLip.

**Figure 2:**
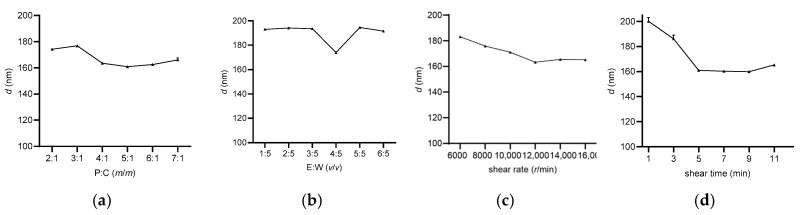
Effect of phospholipid-to-cholesterol ratio (P:C) (**a**), ethanol-to-water ratio (E:W) (**b**), shear rate (**c**), and shear time (**d**) on particle size of Lf-BLips.

To maximize the enrichment of lutein in the brain, it is essential to address its instability and targeting challenges. Liposomes, as amphiphilic carriers with a bilayer membrane structure, were considered to enhance the stability of lutein. Meanwhile, the application of polymers and lactoferrin contributes to improving brain-targeting efficacy (Figure 1). The Lf-LLip delivery system was innovatively prepared using a combination of high-speed shear and ethanol hydration methods. Compared to the ethanol hydration method, this approach reduces particle size without compromising encapsulation efficiency, thereby enhancing the stability of the system. Initially, the BLips were PEGylated, followed by incubation with lactoferrin to facilitate their conjugation to the liposomal bilayer [47]. As shown in Figure 2, the type and concentration of phospholipids influence the structural strength of liposomes [48]. The particle size of the system decreases with increasing egg yolk lecithin concentration in Figure 2a. The study reported by Zhou LL et al. appears a similar trend, though the soybean lecithin concentration used in their study was higher than that of egg yolk lecithin [49]. The ethanol-to-water ratio is crucial for the hydration of the system. Both excessive and insufficient ethanol could lead to a significant increase (*p* < 0.0001) in particle size in Figure 2b. An appropriate amount of ethanol ensures uniform and stable distribution of liposomes in water, obtaining a clear and transparent liposome solution. This study innovatively incorporates high-speed shear. To increase shear speed and time enable the formation of liposomes with smaller particle sizes as seen in Figure 2c,d. Luo et al. also demonstrated that this method significantly enhances the stability of curcumin liposome [50]. Based on the single-factor experiments above, the levels of each factor were determined (Table 2). And subsequent experiments were designed in Table 1.

#### 3.1.2. The CCD-RSM Analysis

**Figure 3:**
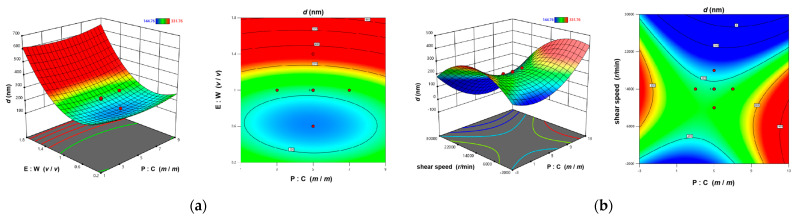

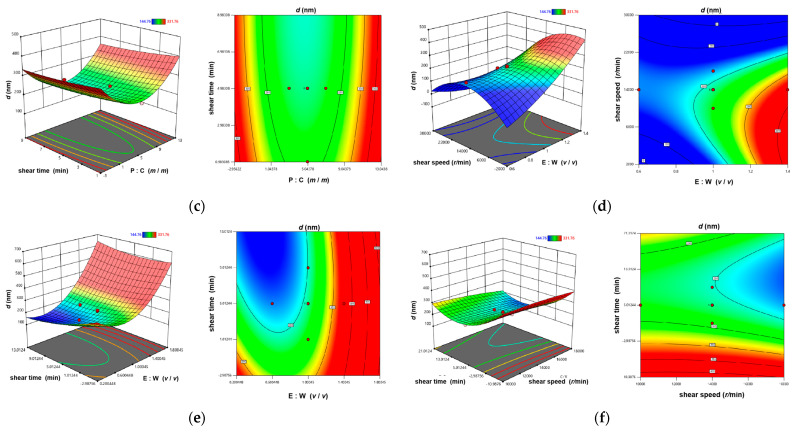
Three-dimensional response surface plots and contour plots showing the interaction effects of various factors: (**a**) phospholipid-to-cholesterol ratio and ethanol-to-water ratio; (**b**) phospholipid-to-cholesterol ratio and shear speed; (**c**) phospholipid-to-cholesterol ratio and shear time; (**d**) ethanol-to-water ratio and shear speed; (**e**) ethanol-to-water ratio and shear time; (**f**) shear speed and shear time.

The results were analyzed by the ANOVA which was performed with the particle sizes as the indicator, yielding the multiple linear regression equation Y = 178.51 − 15.27A − 0.93B + 0.02C − 15.95D + 2.86AB − 0.03AD − 15.95 − 0.01BC + 8.53BD + 2.28A^2^ + 305.85B^2^ + 0.50D^2^. The R^2^ value of equation is 0.9546. The results (Table 3) indicate that the model is significant (*p* < 0.0001) and the lack-of-fit is not significant (*p* > 0.05), demonstrating the reliability of the predictive results. The order of influence of the four factors on Lf-BLip particle size is as follows: B > C > D > A. The 3D response surface plots and contour maps (Figure 3) illustrate the trends of their interactive effects on particle size. The convexity and concavity of the 3D response surface plots reflect the significance of the interaction between two factors [51]. Steeper slopes and more pronounced surface distortions indicate stronger interactive effects. As shown in Figure 3d, the interaction between factors B (phospholipid-to-cholesterol ratio) and C (shear speed) is significant (*p* < 0.05). Additionally, the density of contour lines should be considered [52]. The relatively dense contours in Figure 3c,f indicate that the particle sizes are sensitive to variations in the phospholipid-to-cholesterol ratio, shear speed and shear time.

According to the prediction results and considering the actual equipment conditions, the experimental parameters were determined as follows (shown in Table 4): phospholipid-to-cholesterol ratio of 5:1, ethanol-to-water ratio of 4:5, shear speed of 16,000 rpm, and shear time of 7 min. Through three parallel experimental validations, the particle size of Lf-BLip was measured to be 160.23 ± 2.60 nm, which is essentially consistent with the predicted results.

**Figure 4:**
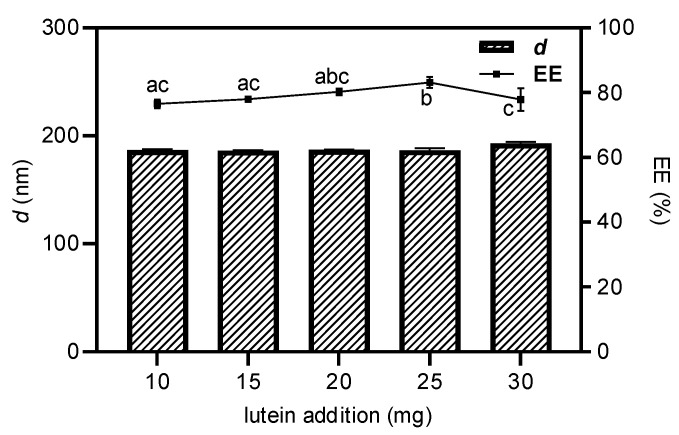
The effect of lutein addition on the particle size and encapsulation efficiency of Lf-LLips. Different letters (a–c) above the bars indicate significant differences at *p* < 0.05 based on one-way ANOVA.

Subsequently, the prepared Lf-BLip was used for lutein loading, and it was necessary to investigate the effect of the lutein addition amount on the system stability. As shown in Figure 4, when the amount of lutein added did not exceed 25 mg, the particle size showed no significant changes (*p* > 0.05). While the encapsulation efficiency gradually increased. This indicates that the loading capacity of liposomes for lutein had not yet reached saturation. However, when 30 mg of lutein was added, the system began to destabilize. Not only was the lutein not effectively encapsulated, but the encapsulation efficiency even exhibited a declining trend. This phenomenon may be attributed to leakage caused by excessive lutein, which was also observed similar trend by Ren et al. during the preparation of proanthocyanidin nanoliposomes [53]. The Lf-LLips prepared under above conditions exhibited an average particle size of 186.63 ± 2.04 nm. Compared with non-PEGylated and lactoferrin-free LLips (59.49 ± 0.51 nm), the incorporation of PEG and lactoferrin resulted in a noticeable increase in liposomal size [54]. The delivery system showed a negative surface charge with a zeta potential of −30.53 ± 1.65 mV and a polydispersity index (PDI) of 0.28 ± 0.01. The junction efficiency of lactoferrin was determined to be 29.78 ± 1.58%. These characters are largely consistent with the lactoferrin–PEG-modified liposomes reported by An et al. [55]. The EE of lutein in Lf-LLips was 83.11 ± 1.67%. Compared with the non-modified LLips (89.53 ± 1.45%), the decrease in the EE value may be attributed to the increased mass proportion of lactoferrin-conjugated PEGylated lipid components, resulting in a relatively lower content of encapsulated lutein at the same volume. This phenomenon aligns with the findings previously reported by Xu et al. [56]. Different PEG chain lengths significantly impact the half-life of liposomes [57]. In this experiment, PEG2000 was shown to extend the systemic circulation time of substances by six-fold or more [58]. Meanwhile, the conjugation of lactoferrin facilitates brain-targeted delivery of lutein.

### 3.2. Morphological Characterization of the Lutein Delivery System

**Figure 5:**
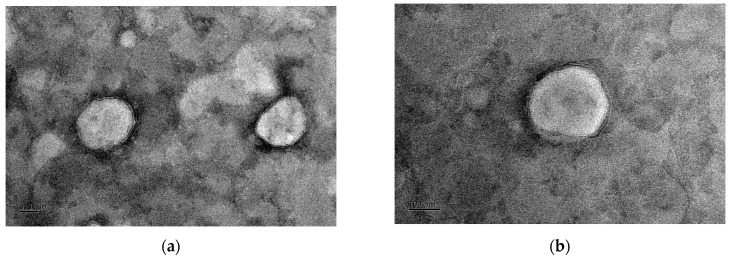
Transmission electron microscopy (TEM) images showing the morphology of LLips and Lf-LLips with a scale bar of 0.1 μm: (**a**) morphology of LLips; (**b**) morphology of Lf-LLips.

The Lf-LLip delivery system appeared as a faint yellow, transparent liquid with a faint blue opalescence when observed at RT. The morphology of Lf-LLips was observed using the TEM, as shown in Figure 5b. Compared with the non-modified LLips exhibited in Figure 5a, the number of LLip particles per unit area at a scale of 0.5 μm was higher than that of Lf-LLips, confirming the larger particle size of Lf-LLips. Both exhibited uniformly dispersed elliptical and spherical structures. A distinct membrane bilayer structure could be clearly observed at the edge of the Lf-LLip particles.

**Figure 6:**
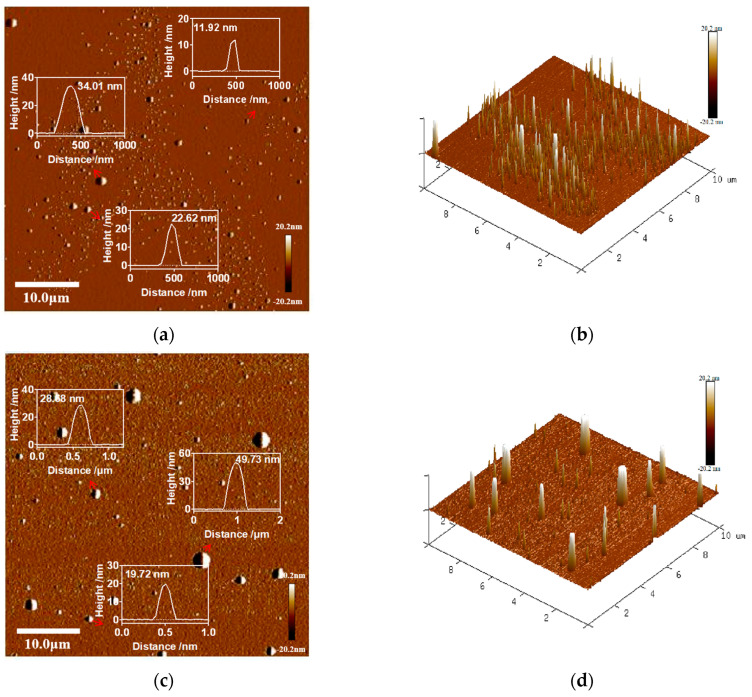
The AFM images showing the nanoscale morphology of LLips and Lf-LLips. (**a**) The height map and various peak force error map (red arrow) of LLips; (**b**) 3D topographic image of LLips; (**c**) The height map and various peak force error map (red arrow) of Lf-LLips; (**d**) 3D topographic image of Lf-LLips.

The nanoscale 2D and 3D morphology of the samples was visualized using the AFM in Figure 6. The images revealed irregularly alternating bright and dark regions, corresponding to protrusions and depressions on the surface. The root mean square roughness (Rq) is widely used to evaluate the surface roughness of nanoparticles and biological membranes [59]. The results indicated that the roughness of Lf-LLips (Rq = 2.7) was higher than that of LLips (Rq = 1.66). This increase in surface roughness may be attributed to the disordered arrangement of PEG and lactoferrin conjugated to the outer layer of the liposomes [58].

### 3.3. FTIR Analysis Results of the Lutein Delivery System

**Figure 7:**
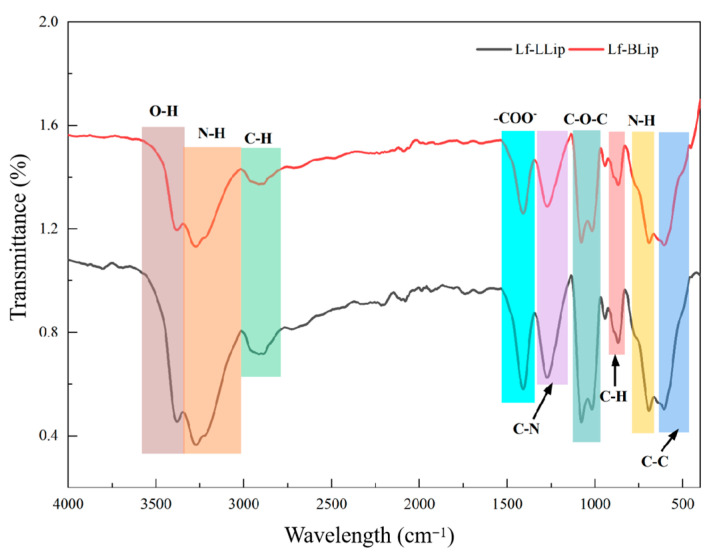
The effect of lutein addition on the particle size and encapsulation efficiency of Lf-LLips.

As shown in Figure 7, Lf-BLip and Lf-LLip exhibit high consistency in their characteristic absorption peaks (3380 cm^−1^, 3268 cm^−1^, 2894 cm^−1^, 1411 cm^−1^, 1270 cm^−1^, 1079 cm^−1^, 1078 cm^−1^, 870 cm^−1^, 691 cm^−1^, 600 cm^−1^). It was confirmed that both possess identical core functional groups and chemical bond structures. It is worth noting that the characteristic peak intensities of the Lf-LLip sample are generally higher than those of Lf-BLip. This difference may stem from the influence of lutein incorporation on molecular dipole moments or hydrogen bonding networks. In the region of 3300–3400 cm^−1^, the broad peak at 3380 cm^−1^ is attributed to the O-H stretching vibration, while the peak at 3268 cm^−1^ corresponds to the N-H stretching vibration of the lactoferrin amide bond (-CONH-) [55]. The presence of these two functional groups indicates strong hydrogen bonding interactions within the system, demonstrating the effective conjugation of lactoferrin. The strong absorption peak at 2894 cm^−1^ originates from the symmetric C-H stretching vibration of both the aliphatic chains (-CH_2_-) and the PEG chains in DSPE-PEG. The peak at 1411 cm^−1^ is assigned to the symmetric stretching vibration of the carboxylate ion (-COO^−^) [60]. The absorption at 1270 cm^−1^ can be attributed to either the C-N stretching vibration of amide III or the P=O stretching vibration of phospholipids. The strong doublet at 1079 cm^−1^ and 1078 cm^−1^ represents the characteristic absorption of the ether linkage (C-O-C) stretching vibration in PEG chains. The weak peak at 870 cm^−1^ may arise from the C-H out-of-plane bending vibration of aromatic amino acids (such as tryptophan) in lactoferrin. The absorption at 691 cm^−1^ is likely derived from the N-H out-of-plane bending vibration of amide V. The absorption near 600 cm^−1^ belongs to complex vibrations in the fingerprint region, specifically corresponding to C-C bending vibrations.

### 3.4. Stability of Lactoferrin-Modified Lutein Liposomes (Lf-LLips)

Lutein possesses multiple conjugated double bonds and exists as both all-trans and cis-conformational isomers, with the all-trans form being the most stable. At elevated temperatures, lutein can undergo isomerization to cis-isomers or form epoxides, resulting in reduced biological activity. Exposure to light, particularly ultraviolet radiation, accelerates the oxidation of lutein. It leads to the degradation of its chromophore structure and consequent loss of color and functionality. Previous studies have demonstrated that liposomal encapsulation can effectively protect sensitive nutrients from environmental degradation [61], thereby preserving their bioactivity. The stability of Lf-LLips was systematically evaluated under controlled temperature and light conditions over a 7-day period, with the values of the particle size (*d*) and lutein retention rate (RR) as key indicators. Additionally, a 28-day storage study was conducted to establish the shelf-life profile and ensure the long-term stability of the formulation.

#### 3.4.1. Effect of Light on the Stability of Lf-LLips

**Figure 8:**
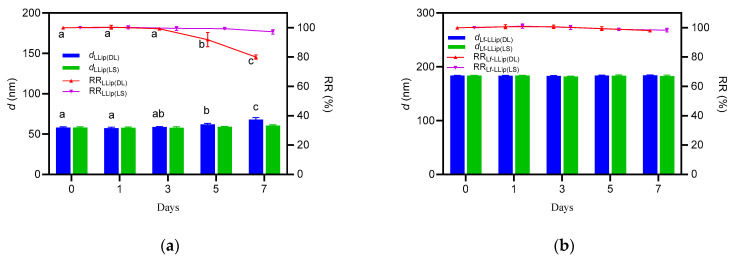
The effect of light on the particle size and lutein retention rate of LLips and Lf-LLips: (**a**) particle size and lutein retention rate of LLips. Different letters (a–c) above the bars indicate significant differences at *p* < 0.05 based on one-way ANOVA; (**b**) particle size and lutein retention rate of Lf-LLips.

Due to its photosensitivity, lutein is highly susceptible to light. Previous studies have confirmed that ultraviolet radiation can rapidly decompose lutein [62]. To simulate application conditions during processing, the *d* value and RR of lutein in both LLips and Lf-LLips were evaluated at room temperature with diffusion light (DL) and light shielding (LS). The results over a 7-day period are shown in Figure 8. Both LLips and Lf-LLips maintained stable particle sizes and retention rates within the first 3 days. The *d* values of LLips began to fluctuate up to day 5, and the RR of lutein dropped to approximately 80% by day 7. In contrast, Lf-LLips exhibited almost no significant changes throughout the experimental period. The study demonstrates that the PEGylated bilayer liposomal structure can effectively protect lutein from photodegradation.

#### 3.4.2. Effect of Temperature on the Stability of Lf-LLip

Generally, samples were stored at 4 °C and room temperature (RT, 25 ± 2 °C) during the experimental process. Additionally, the PEGylation procedure required a water bath at 50 °C. Therefore, the changes in the *d* value and RR of lutein were evaluated at 4 °C, RT, and 50 °C.

**Figure 9:**
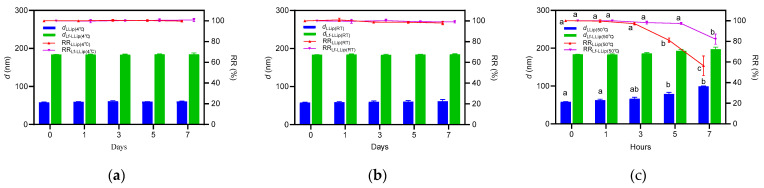
The effect of temperature on the particle size and lutein retention rate of LLips and Lf-LLips: (**a**) particle size and lutein retention rate of LLips and Lf-LLips at 4 °C; (**b**) particle size and lutein retention rate of LLips and Lf-LLips at RT; (**c**) particle size and lutein retention rate of LLips and Lf-LLips at 50 °C. Different letters (a–c) above the bars indicate significant differences at *p* < 0.05 based on one-way ANOVA.

Figure 9a,b show that, when stored at 4 °C or RT, both LLips and Lf-LLips showed almost no fluctuation in particle size over 7 days. No significant changes in the lutein retention rate were observed within the first 3 days (*p* > 0.05). Compared with LLips, Lf-LLips kept the EE of lutein above 85% after 7 days. It is evident that sealing and light-shielding storage at 4 °C or RT can substantially extend the shelf life of lutein. Moreover, the bilayer structure of the liposomes and the surrounding polymer chains provided enhanced encapsulation of lutein, further improving its stability.

When stored under high-temperature conditions (50 °C), lutein degraded rapidly. The results shown in Figure 9c demonstrate that both formulations underwent notable changes within just 7 h. The particle size increased to over 400 nm, and the RR of lutein decreased by 43.61% and 17.91% for LLips and Lf-LLips, respectively. The LLip solution also became turbid. This instability may be because heat-induced denaturation of lactoferrin, leading to structural disruption of the delivery system [63]. The degradation of lutein was accelerated subsequent to its leakage. At 40 °C, a significant decrease in the retention rate (RR) of commercially available lutein crystals (≥75%) was also observed within 10 days. Therefore, it is recommended to avoid exposing lutein to high temperatures, and the duration of the 50 °C water bath during the preparation process should not exceed 3 h.

#### 3.4.3. Effect of Storage Duration on the Stability of Lf-LLips

**Figure 10:**
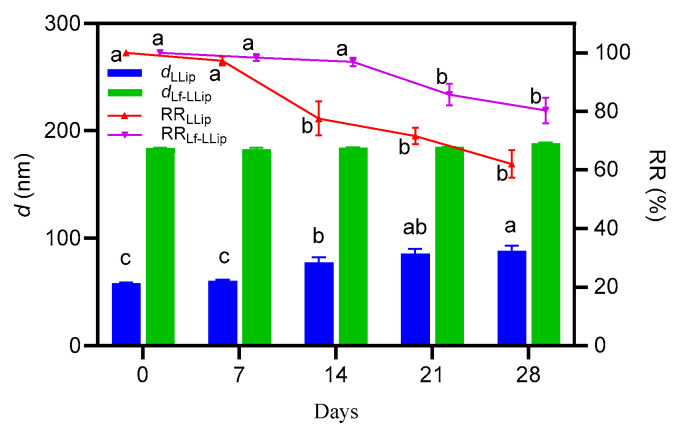
The effect of storage duration on the particle size and lutein retention rate of LLips and Lf-LLips. Different letters (a–c) above the bars indicate significant differences at *p* < 0.05 based on one-way ANOVA.

Following the analysis of the effects of light and temperature on the stability of the delivery system, it was determined that Lf-LLips should be stored at 4 °C under light-shielding conditions. To further investigate its stability, changes in the *d* values and RR of lutein in the system over a 28-day period at the above conditions were evaluated, as shown in Figure 10.

Although both formulations exhibited an increase in particle size over time, compared with LLips, the *d* values of Lf-LLips showed no significant changes (*p* > 0.05) and maintained a lutein retention rate of approximately 80% at day 28. The observed growth in particle size and the decrease in lutein retention may be attributed to liposomal sedimentation. The surface tension of liposomes decreases, increasing cholesterol concentration [64]. Furthermore, it cannot be ruled out that partial degradation of lipid components may have compromised the integrity of the membrane structure, leading to dislodging of lactoferrin or polymer chains. These processes could promote the formation of large aggregates and result in the leakage of loaded lutein. Considering the above factors, lyophilization or the addition of suitable preservatives may be employed to enhance the stability of Lf-LLips for extended storage periods.

### 3.5. In Vitro Release Characteristics of Lf-LLips

**Figure 11:**
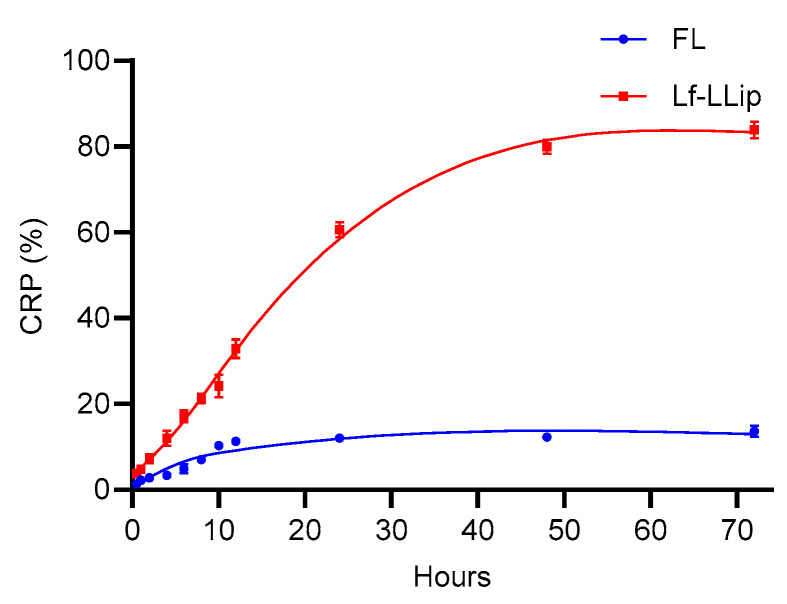
The effect of storage duration on the particle size and lutein retention rate of LLips and Lf-LLips.

The ability to achieve a sustained-release profile is critical for a nutrient delivery system. Sustained release enables the gradual and controlled release of nutrients in the body, which maximizes their biological activity in contrast to burst release. The cumulative release characteristics of Lf-LLips over 72 h and the corresponding release model are shown in Figure 11 and Table 1. The results indicated that the initial cumulative release percentage (CRP) of Lf-LLips and free lutein (FL) were similar. During the period of 12 to 24 h, the CRP of Lf-LLips reached approximately 60%, while that of FL was still only about 10%. By the end of 72 h, the CRP of Lf-LLips was 6.17 times higher than that of FL. The in vitro release curves were fitted using the zero-order kinetics model, first-order kinetics model, Higuchi equation model, Ritger–Peppas equation model, and Weibull distribution model. Ritger–Peppas equation model must strictly adhere to data points where the cumulative release rate is lower than 60%.

As shown in Table 5, the curve of FL complied with the first-order kinetics model (R^2^ = 0.9880). The Weibull model is widely used to describe the release kinetics of substances from complex delivery systems, such as those involving polymer erosion [65]. The results indicate that the release behavior of sLf-LLip follows a diffusion–swelling/erosion mechanism. The study demonstrates that the developed Lf-LLip delivery system effectively achieves the sustained release of lutein, offering a promising strategy for enhancing its bioavailability.

### 3.6. Brain-Targeting Evaluation of Lf-LLips

The blood–brain barrier (BBB) is a protective structure centered around brain microvascular endothelial cells, which, together with pericytes, astrocytes, and microglia, form the neurovascular unit. It establishes a tightly interconnected barrier through tight junctions [66]. With the exception of small-molecule lipophilic substances [67], virtually no other compounds can penetrate this barrier. Compared with monoculture-based BBB models, co-culture models offer significant advantages for studying substance permeability across the BBB [68]. Based on the model establishment methods described by Jun Sung Park et al. [69], the present study employed a co-culture system consisting of bEnd.3 cells (murine brain microvascular endothelial cells) and C8D1A cells (murine cerebellar astrocytes) to simulate the BBB. The formation of the BBB model was monitored by measuring the transepithelial electrical resistance (TEER).

#### 3.6.1. Cell Cytotoxicity

**Figure 12:**
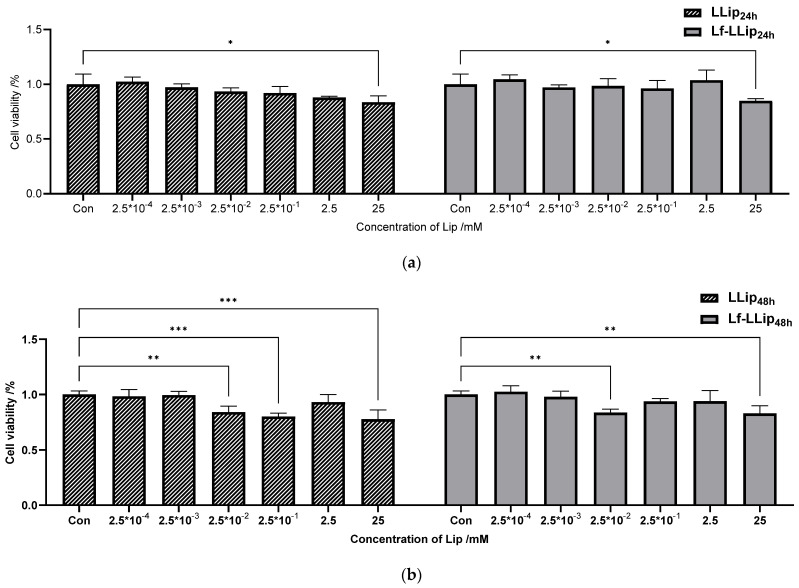

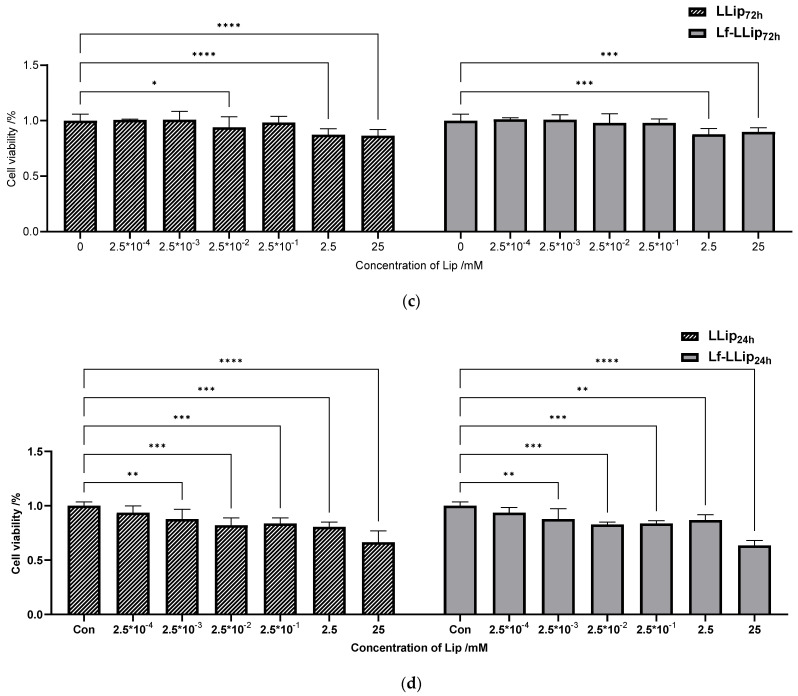

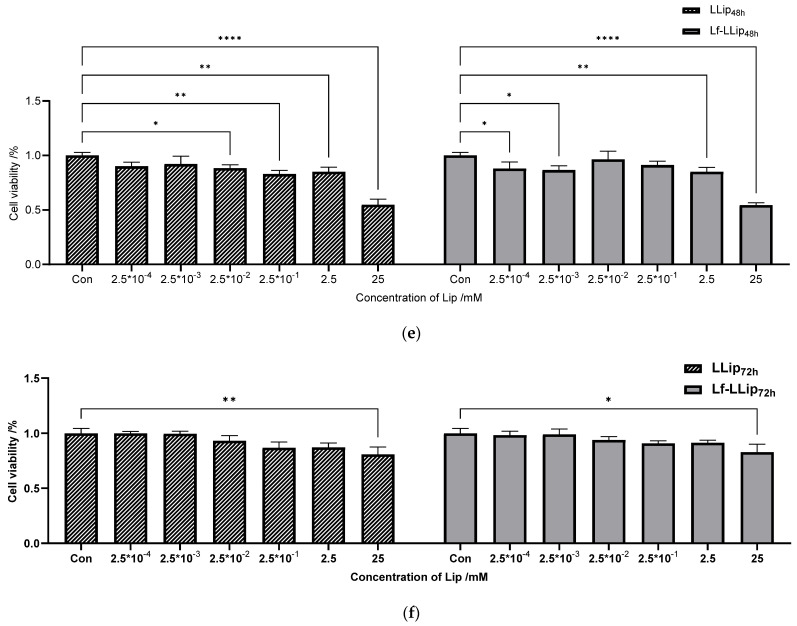
The effects of LLips and Lf-LLips on the cell viability of bEnd.3 and C8D1A cells at 24, 48, and 72 h: (**a**) effects of LLips and Lf-LLips on the cell viability of bEnd.3 cells at 24 h; (**b**) effects of LLips and Lf-LLips on the cell viability of bEnd.3 cells at 48 h; (**c**) effects of LLips and Lf-LLips on the cell viability of bEnd.3 cells at 72 h; (**d**) effects of LLips and Lf-LLips on the cell viability of C8D1A cells at 24 h; (**e**) effects of LLips and Lf-LLips on the cell viability of C8D1A cells at 48 h; (**f**) effects of LLips and Lf-LLips on the cell viability of C8D1A cells at 72 h. * means *p* < 0.05; ** means *p* < 0.01; *** means *p* < 0.001; **** means *p* < 0.0001.

Cell cytotoxicity is a main factor in evaluating targeted delivery systems. The effects of Lf-LLips on the viability of bEnd.3 and C8D1A cells at 24, 48, and 72 h are presented in Figure 12. Overall, Lf-LLips exhibited lower cytotoxicity toward both cell types compared with LLips. However, both liposomes had a more pronounced effect on the viability of C8D1A cells. Figure 12a–c illustrate the cell viability of bEnd.3 cells following treatment with LLips and Lf-LLips. The difference became more pronounced after 72 h (*p* < 0.0001). The viability of C8D1A cells under the same experimental conditions is shown in Figure 12d–f. Only the highest concentration (25 mM) resulted in a significant reduction in viability (*p*_LLip_ < 0.01, *p*_Lf-LLip_ < 0.05) at 72 h. Interestingly, this trend contrasts with bEnd.3 cells in 72 h, suggesting that C8D1A cells may be more sensitive to both LLips and Lf-LLips. Within the first 24 h and across the concentration range of 2.5 × 10^−4^ to 2.5 µM, the systems maintained cell viability above 85% in both bEnd.3 and C8D1A cells, indicating negligible cytotoxicity. Thus, a concentration of 2.5 µM was chosen for subsequent blood–brain barrier (BBB) permeability experiments to ensure the optimal efficacy. Studies have indicated that cationic liposomes typically exhibit higher cytotoxicity [70], while anionic liposomes demonstrate limited toxicity or are relatively safe [71]. No cytotoxicity observed in the Lf-LLips prepared in this study may be attributed to PEGylation. Setyawati et al. reported that incorporating cholesterol and PEGylated lipids into cationic liposomes reduced their cytotoxicity, even increasing cell survival rates to as high as 122.67% [72]. Furthermore, several other studies have confirmed that PEGylated liposomes not only exert a protective effect on normal cells or tissues, but also enable the targeted and efficient delivery of antitumor drugs to eliminate harmful cells [73,74,75].

#### 3.6.2. The Permeability Across the BBB

**Figure 13:**
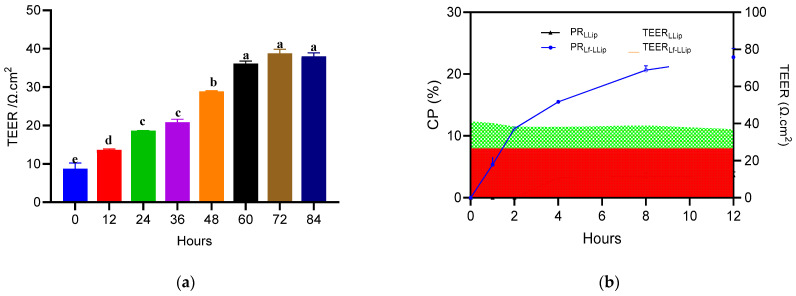
The monitoring of TEER values in the BBB model and the permeability of LLips and Lf-LLips at 12 h: (**a**) TEER values in the BBB model at 84 h. Different letters (a–e) above the bars indicate significant differences at *p* < 0.05 based on one-way ANOVA; (**b**) TEER values and permeability trends of LLips and Lf-LLips across the BBB.

A complete medium solution containing LLips or Lf-LLips at a concentration of 2.5 mM was added to the upper chamber of a Transwell system. Samples were collected from the lower chamber at 1, 2, 4, 8, and 12 h, while the transepithelial electrical resistance (TEER) was monitored simultaneously. The permeability was measured and calculated, as shown in Figure 13. LLips exhibited only minimal permeability starting at 4 h, which may be attributed to transient transcellular diffusion [9]. Although small-molecule lipophilic compounds can potentially enter the brain via this route, they may become trapped within the cell membrane, resulting in an effective permeability of zero [76]. The permeability of Lf-LLips at 12 h was 22.73 ± 1.42%. Studies have shown that polyethylene glycol (PEG), with a molecular weight of 2 kDa, exhibits enhanced cellular uptake efficiency [77]. The PEGylation of Lf-LLips prolongs their circulation time in the bloodstream, thereby promoting more effective accumulation in the brain [67,78]. Moreover, the conjugated lactoferrin can bind to lactoferrin receptors (LfRs) in the brain [79], thereby enhancing the permeability efficiency of lutein. Multiple studies have confirmed that this structural modification improves BBB penetration for various nutrients and neurotrophic drugs [80,81,82,83].

## 4. Conclusions

This study developed and characterized PEGylated lutein liposomes conjugated with lactoferrin (Lf-LLips) with a particle size of 186.63 ± 2.04 nm, a potential of −30.53 ± 1.65 mV and a PDI of 0.28 ± 0.01. The Encapsulation Efficiency of Lf-LLips was 83.11 ± 1.67%. The morphology of Lf-LLips was observed as spheroidal. It exhibited good stability at various temperatures under different light conditions compared to LLip. And storage durations was investigated in 28 days, the retention rate of lutein remained above 80%. The in vitro release characters demonstrated desirable sustained-release properties. Moreover, Lf-LLips successfully penetrated the blood–brain barrier, enabling effective brain-targeted delivery. Future work will involve animal studies to evaluate their therapeutic effects on a mouse model of brain aging and explore the underlying mechanisms. This study provides a scientific basis for the development and application of lutein-related functional foods and special dietary products.

## Figures and Tables

**Table 1:** CCD–RSM Experiment.

NO.	P:C	E:W	Shear Speed	Shear Time
1	0	0	−2	0
2	−1	1	1	−1
3	1	−1	1	−1
4	−1	1	−1	−1
5	1	−1	−1	−1
6	0	0	0	2
7	−1	1	1	1
8	1	1	1	1
9	−1	−1	1	1
10	−1	−1	1	−1
11	0	0	0	0
12	−1	−1	−1	1
13	0	−2	0	0
14	−1	−1	−1	−1
15	0	0	0	0
16	1	−1	1	1
17	0	0	0	0
18	−1	1	−1	1
19	0	0	0	0
20	1	1	−1	−1
21	0	0	0	−2
22	1	−1	−1	1
23	−2	0	0	0
24	0	0	2	0
25	1	1	1	−1
26	0	0	0	0
27	2	0	0	0
28	0	2	0	0
29	1	1	−1	1
30	0	0	0	0

**Table 2:** Factors and their levels for CCD-RSM experiments.

Factors	Levels			
	Low (−1)	High (1)	−alpha	+alpha
A—P:C (*m*/*m*)	4	6	3	7
B—E:W (*v*/*v*)	0.8	1.2	0.6	1.4
C—shear speed (r/min)	12,000	16,000	10,000	18,000
D—shear time (min)	3	7	1	9

**Table 3:** Results of analysis of variance.

Source	Sum of Squares	df	Mean Square	F-Value	*p*-Value	Significant
Model	63,820.32	14	4558.59	22.52	<0.0001	Y
A-P:C	68.41	1	68.41	0.3379	0.5697	N
B-E:W	51,307.9	1	51,307.9	253.44	<0.0001	Y
C-V	4241.11	1	4241.11	20.95	0.0004	Y
D-T	2265.93	1	2265.93	11.19	0.0044	Y
AB	5.24	1	5.24	0.0259	0.8743	N
AC	46.38	1	46.38	0.2291	0.6391	N
AD	0.0625	1	0.0625	0.0003	0.9862	N
BC	1009.33	1	1009.33	4.99	0.0412	Y
BD	186.32	1	186.32	0.9204	0.3526	N
CD	13.62	1	13.62	0.0673	0.7989	N
A^2^	142.17	1	142.17	0.7023	0.4152	N
B^2^	4105.37	1	4105.37	20.28	0.0004	Y
C^2^	182.69	1	182.69	0.9024	0.3572	N
D^2^	110.72	1	110.72	0.5469	0.471	N
Residual	3036.68	15	202.45			
Lack of Fit	2688.01	10	268.8	3.85	0.0747	N
Pure Error	348.67	5	69.73			
Cor Total	66,857	29				

Note: “Y” has a significant effect; “N” has no significant effect.

**Table 4:** Validation experiment and results.

Experiment Number	A—P:C	B—E:W	C—Shear Speed	D—Shear Time	*d* (nm)
NO.1	5:1 (m/m)	4:5 (*v*/*v*)	16,000 r/min	7 min	163.17
NO.2					158.22
NO.3					159.31
Average of *d* value					160.23 ± 2.60

**Table 5:** Construction of release kinetics models for FL and Lf-LLips.

Model Name	Model Equation	Fitting Equation of FL	Fitting Equation of Lf-LLips
Zero-Order Kinetics Model	y = a + bx	y = 5.1976 + 1.3788x R^2^ = 0.9061	y = 4.1664 + 0.1849x R^2^ = 0.5805
First-Order Kinetics Model	y = a × (1 − e^−bx^)	y = 93.1526 × (1 − e^−0.0377x^) R^2^ = 0.9880	y = 12.7943 × (1 − e^−0.1226x^) R^2^ = 0.9469
Higuchi Equation Model	y = a × (x^1/2) + b	y = 11.3368x^1/2^ − 5.9282 R^2^ = 0.9674	y = 1.7765x^1/2^ − 1.3442 R^2^ = 0.7865
Ritger–Peppas Equation Model	y = a × (x^b)	y = 6.0674x^0.6439^ (CRP ≤ 60%) R^2^ = 0.8012	y = 3.2277x^0.3660^ (CRP ≤ 60%) R^2^ = 0.9909
Weibull Distribution Model	y = a − (a − b) × e^−(kx)^d^	y = 12.3933 − 10.5388e^(−0.1146x)^2.5986^ R^2^ = 0.9867	y = 83.8645 − 80.3023e^(−0.0474x)^1.3967^ R^2^ = 0.9989

## Data Availability

The original contributions presented in the study are included in the Article; further inquiries can be directed to the first author or the corresponding author.
